# Knowledge and Demand for Information about Islet Transplantation in Patients with Type 1 Diabetes

**DOI:** 10.1155/2011/136298

**Published:** 2011-11-22

**Authors:** Yuko Yamamoto, Masakazu Nishigaki, Naoko Kato, Michio Hayashi, Teruo Shiba, Yasumichi Mori, Tetsuro Kobayashi, Keiko Kazuma

**Affiliations:** ^1^Department of Adult Nursing, Graduate School of Medicine, School of Health Sciences and Nursing, The University of Tokyo, 7-3-1 Hongo, Bunkyo-ku, Tokyo 113-0033, Japan; ^2^Department of Diabetes and Endocrinology, Kanto Medical Center NTT EC, Tokyo 141-8625, Japan; ^3^Department of Diabetes and Endocrinology, Mitsui Memorial Hospital, Tokyo 101-8643, Japan; ^4^Department of Endocrinology and Metabolism, Toranomon Hospital, Tokyo 105-8470, Japan; ^5^Third Department of Internal Medicine, University of Yamanashi, Yamanashi 409-3898, Japan

## Abstract

This cross-sectional study based on self-administrated questionnaire was conducted to investigate knowledge, related factors, and sources of information regarding islet transplantation in patients with type 1 diabetes in Japan. Among 137 patients who provided valid responses, 67 (48.9%) knew about islet transplantation. Their main source of information was newspapers or magazines (56.7%) and television or radio (46.3%). However, 85.8% of patients preferred the attending physician as their source of information. Although more than half of the patients were correctly aware of issues related to islet transplantation, the following specific issues for islet transplantation were not understood or considered, and there was little knowledge of them: need for immunosuppressants, lifestyle and dietary adaptations, fewer bodily burdens, and complications. The experience of hypoglycaemia, a high level of academic background, frequent self-monitoring of blood glucose, and the use of continuous subcutaneous insulin infusion were related to higher knowledge about islet transplantation.

## 1. Introduction

As an alternative to intensive insulin injection therapy, pancreatic transplantation and islet transplantation are being established hoping for a radical cure for patients who are at serious risk of poor glycemic control. Although pancreatic transplantation could cure type 1 diabetes, its outcomes are not necessarily favorable than conventional therapy, due to invasive surgical procedure and/or toxicity of immunosuppression procedure [1, 2]. Islet transplantation once dramatically progressed by the establishment of the Edmonton protocol [3]. Initially, short-term effects by islet transplantation had demonstrated that it achieved a highly improved rate of temporal insulin independency [3]. But original Edmonton protocol with nondepleting anti-T-cell antibody did not achieve longitudinal insulin independency [4]. So the current islet transplantation protocol needs an active immunosuppression procedure, though it is still a less “surgically” invasive procedure than pancreatic transplantation [5–7]. In spite of these limited effects and physical burden, patients with islet transplantation report less concern about hypoglycaemia [8, 9], which is one of the most significant factors associated with quality of life in patients with type 1 diabetes. This study suggests that islet transplantation should improve quality of life of type 1 diabetic patients, though its actual improvement remains unclear because of small sample size [10].

Islet transplantation in Japan is conducted from nonheart-beating donors because there are few heart-beating brain-dead donors in Japan per year and their pancreata are used for pancreas transplantation. The donation procedure from a nonheart-beating donor is more complicated than that from a brain dead donor [11]. Pancreatic islet transplantation has been conducted 34 times in 18 patients in Japan [12] while 649 times in 325 patients between 1999 to 2007 in western countries according to the Collaborative Islet Transplant Registry [13]. 

Although the donor shortage and technical issues limit the application of islet transplant at present, the dramatic progress of procedures may solve the limitations: improvement in methods for the isolation and preservation of islet cells [14, 15], xenotransplantation or reproduction islet transplantation [16, 17], and no requirement for multiple donors [18]. Moreover, a recent immunosuppressive procedure that is now undergoing a phase III trial [19] and the use of glucagon-like peptide-1 receptor agonist after islet transplantation [20, 21] show improved beta-cell function and prolonged insulin independence. These progressive findings may encourage patients and medical professionals who consider application of islet transplantation, and then islet transplantation may be generally available for the medical treatment of type 1 diabetes in Japan in the near future. Taking this into account, it is necessary to prepare a system for responding to forthcoming patient needs about islet transplantation.

Previous research shows that posttransplantation patients often find it difficult to bear the need for stricter restriction of their dietary habits and physical activity than during the pretransplantation period in order to maintain control of their blood glucose level [22]. In addition, posttransplantation patients need to ensure adequate dietary behavior in order to avoid overloading of the grafted islets, which is unnecessary in pancreas transplantation [23]. This sense of bearing a burden may be caused by the gap between reality and the patient's pre-transplant idea of daily life after transplantation; patients might think that they will no longer be restricted in their lifestyle habits by diet therapy, exercise therapy, and insulin injections. In order to resolve the problem presented by this gap, it is necessary to understand the patient's knowledge and perceptions related to treatment and supply accurate information when medical professionals discuss options for medical treatment with patients [24]. It is also important to supply information that the patient requires and supply it from suitable sources in order to improve its acceptability to patients.

In the present study, a self-administered questionnaire survey was provided to type 1 diabetes patients to clarify (1) their knowledge of islet transplantation and (2) actual and desired information sources about islet transplantation.

## 2. Methods

### 2.1. Setting and Participants

This cross-sectional observational study based on a self-administered questionnaire was conducted at the diabetes clinic of four general hospital in 2008 July to November type 1 diabetes patients who were being treated at the diabetes clinic were enrolled. The inclusion criteria were as follows: (1) age between 20 and 75 years and (2) at least 6 months since diagnosis with type 1 diabetes. Patients not fluent in Japanese or with cognitive impairment were excluded.

When patients visited the clinic, a clinician assessed whether they met the inclusion criteria. The nature of the study was explained to eligible patients by one of the investigators. For ethical reasons, it is unavoidable to make reference about islet transplantation when research associates explain this study to patients and obtain consent. So a simple outline of islet transplantation was therefore given to all participants. After providing written informed consent to participate in the study, each patient was asked to complete a questionnaire. The questionnaire was collected immediately after the patient completed it.

### 2.2. Questionnaire

The content validity and face validity of the questionnaire were confirmed by a panel of experts in each specialist area: islet transplantation, treatment, nursing, and research using questionnaires. The following questionnaire items were derived from existing guidelines about islet transplantation or generated from existing literature by the authors. Face validity and content validity were confirmed by pretest in 6 patients with type 1 diabetes. 

Patients were asked whether they were aware of the term “islet transplantation” before their participation in this study. Patients who answered that they did know the term were then asked about their detailed perceptions by asking them whether they agreed or disagreed with the 10 statements about islet transplantation (Table 3). Some of the statements were not necessarily correct. The patients were asked to answer the items on a four-point Likert scale (“agree,” “moderately agree,” “moderately disagree,” “disagree”), or to respond “Do not know.”

Patients who answered that they knew about islet transplantation were asked where they obtained information about it. All patients were then asked whether they wanted information about islet transplantation. Those who replied that they wanted information were then asked which kind of information source they preferred. The questions about information source were both in multiple-choice form: attending physician, other physicians, other medical professionals, family, patients with type 1 diabetes or patient society, newspaper/magazine, TV/radio, and the Internet.

The following variables were investigated as subjects' clinical characteristics: body mass index (BMI, kg/m^2^), duration of diabetes (years), current treatment (insulin self-injection (yes/no), continuous subcutaneous insulin infusion (CSII) (yes/no)), frequency of insulin injection, and self-monitoring of blood glucose (SMBG). The following symptom-related characteristics were investigated: whether the patient had complications related to diabetes (retinopathy, nephropathy, neuropathy (yes/no)); frequency and details of hypoglycaemia in the last 4 weeks. Other background characteristics investigated were as follows: gender, age, educational status, living status, marital status, occupational status, and social support.

### 2.3. Statistical Analysis

First, descriptive statistics for each variable were tabulated. Next, univariate logistic regression analysis was performed to assess which factors affected the knowledge of islet transplantation (“know” was scored 1; “do not know” was scored 0). Subject characteristics were treated as independent variables. SAS version 9.13 software (SAS Institute, Cary, NC, USA) was used for statistical analysis, and the level of significance was set at *P* < 0.05.

### 2.4. Results

Of the 143 eligible patients who visited the clinic during the study period, 137 (95.8%) completed the questionnaire.  Table 1 shows the characteristics of the subjects.

Sixty-seven (48.9%) patients answered that they knew the term “islet transplantation.” Table 2 shows the actual and desired sources of information. Of the 137 subjects, 120 (87.6%) patients answered that they wanted information about islet transplantation. Patients used newspapers/magazines (56.7%) and TV/radio (46.2%) as information sources most frequently. On the other hand, the “attending physician” was the most desired information source (85.8%).

Table 3 shows knowledge and detailed perceptions of islet transplantation. Among the correct statements, more than half of the patients answered “agree”/“moderately agree” to the following items: “islet transplantation stabilizes blood sugar level”; “islet transplantation is expensive”; “there are few donors in Japan.” For the items “islet transplantation causes fewer physical burdens” and “islet transplantation is conducted mainly in a person having difficulty achieving glycemic control” the answer tended to be “disagree”/“moderately disagree” (38.8% and 47.7%, resp.). About half of the patients answered “do not know” to the items “complication of islet transplantation is severe,” “islet transplantation makes immunosuppressant indispensable” (53.7% and 44.8%, resp.).

Table 4 shows the relationship between the knowledge of islet transplantation and patients' characteristics. Variables that showed significant association with a good level of knowledge were high educational status (odds ratio (OR) = 2.45 (95% CI 1.23–4.88)), frequent SMBG (OR = 1.26 (1.04–1.52)), and experience of hypoglycemic events within the last 4 weeks (OR = 4.66 (1.75–12.4)).

## 3. Discussion

The HbA1C level, BMI, and other demographic characteristics in the subjects of this study were similar to those in a previous nationwide survey [25]. So results in this study can be regarded as reflecting the common sense of Japanese patients with type 1 diabetes. Almost half of the subjects in this study knew about islet transplantation. The sources of information of the patients who knew about pancreatic islet transplantation were mainly the mass media, but they regarded their attending physician as the most acceptable information source. Little information about islet transplantation is offered by medical professionals at present. Even if patients are interested in islet transplantation, it is not easy for them to obtain valid information from medical professionals. It is to be expected that physicians have difficulty in supplying information because islet transplantation is still a special treatment, and even attending physicians do not have sufficient information. Since the mass media disseminate population-based information, it is not necessarily appropriate for individual patients. It is often hard for patients to know what is appropriate information and sometimes they might choose to accept inappropriate information [26]. There is thus an unmet need that should be resolved in order to prevent inappropriate understanding of islet transplantation. 

In spite of this information shortage, patients have appropriate perceptions of the expense of islet transplantation and the shortage of donors. However, these are common problems not only in islet transplantation but also in the transplantation of organs generally, and this is probably widely understood. On the other hand, the perception of items specific to islet transplantation was different. Prognosis-related items related to glycemic stability and insulin-free status [27] were well understood whereas the treatment advantage of fewer physical burdens was less well understood [3]. Furthermore, disadvantages and complications of islet transplantation tend to be unrecognized. This shows that patients who had partial knowledge about islet transplantation were biased toward the idea of a favorable treatment outcome. It is necessary to provide patients with more information about the advantages and disadvantages of the islet transplantation procedure to enable them to arrive at a valid, informed choice. Especially, medical professionals must explain carefully that islet transplantation is a developing procedure, with insufficient information about clinical outcome [28].

Experience of hypoglycaemia and frequent SMBG were related to good knowledge of islet transplantation. Hypoglycemic events lead to discouragement in the daily life of patients [29]. Frequent SMBG is also a burden for patients. In particular, the burden of SMBG would be more severe in patients with poor glycemic control because the frequency of SMBG increases as glycemic control worsens [30]. Thus, patients would desire radical treatment because their quality of life is threatened by hypoglycaemia or frequent SMBG due to poor glycemic control. The relationship between educational status, CSII usage, and knowledge might indicate that health literacy and the decision to seek new treatment would also motivate patients to seek information about islet transplantation.

Some of the limitations of this study will now be discussed. First, whether these findings are specific to Japanese type 1 diabetic patients remains unclear, because this study did not contain a control group of nondiabetic adults. Second, as mentioned in Section 2, we gave brief information about islet transplantation to the patients when we obtained their informed consent. Although the information given to them consisted only of a simple outline of the islet transplantation procedure, there might have been some effects on the patients' responses, especially to the question about their detailed perceptions. Additionally, since the information was brief, subjects should answer the question with ambiguous knowledge about islet transplantation. For example, if the information about requirement for multiple donors was given to the subjects, they could have negative impression towards islet transplantation.

Despite these weaknesses, this is the first study that clarified knowledge, detailed perceptions, and information sources regarding islet transplantation in type 1 diabetes patients in Japan. These results may provide a valuable source of reference for medical professionals offering patients islet transplantation as a treatment option.

## Figures and Tables

**Table 1:** Subject characteristics (*N* = 137).

	Number (%), mean ± SD
Gender (male)	76 (55.5)
Age (years)	52.0 ± 12.7
BMI (kg/m^2^)^(1)^	21.9 ± 11.4
Educational status	
Junior high school	9 (6.6)
High school	60 (43.8)
Junior college	24 (17.5)
University	43 (31.4)
Occupation status	
Full time	70 (51.1)
Part time	19 (13.9)
None	42 (30.7)
Others	5 ( 3.6)
Living with others	111 (81.0)
Married	100 (73.0)
Onset of diabetes (age)	38.2 ± 14.8
Duration of diabetes (years)	13.7 ± 10.8
Diabetic complication	
Neuropathy	36 (26.3)
Retinopathy	37 (27.0)
Nephropathy	17 (12.4)
Current treatment	
Insulin injections	124 (90.5)
CSII^(2)^	13 ( 9.5)
HbA1C (NGSP, %)	7.9 ± 1.2
SMBG (times)	3.0 ± 1.8
Hypoglycemic episode (yes)	109 (79.6)
Hypoglycemic episode (times)	5.2 ± 4.5

Continuation values are means ± SD.

^(1)^Body mass index.

^(2)^Continuous subcutaneous insulin infusion.

**Table 2:** Actual/desired information sources islet transplantation.

	Actual^(1)^ *n* = 67	Desired^(2)^ *n* = 120
	*n* (%)	*n* (%)
Attending physician	9 (13.4)	103 (85.8)
Other physician	5 (7.5)	35 (29.4)
Other medical professionals	2 (3.0)	32 (26.7)
Family	2 (3.0)	2 (1.7)
Other T1DM patients or patient society	6 (9.0)	22 (18.3)
Newspaper/magazine	38 (56.7)	43 (35.8)
TV/radio	31 (46.2)	31 (25.8)
Internet	17 (25.4)	36 (30.0)

^(1)^Only the subjects who answered that they knew islet transplantation.

^(2)^Only the subjects who answered that they required information about islet transplantation.

**Table 3:** Detailed perception of islet transplantation (*N* = 67).

	Agree	Moderately agree	Moderately disagree	Disagree	Do not know
	*n* (%)	*n* (%)	*n* (%)	*n* (%)	*n* (%)
Stabilization of blood sugar level	29 (43.3)	29 (43.3)	2 (3.0)	1 (1.5)	6 (9.0)
Its expensive	39 (58.2)	9 (13.4)	1 (1.5)	0 (0.0)	18 (26.9)
There are few donors	33 (49.3)	10 (14.9)	2 (3.0)	1 (1.5)	21 (31.3)
Fewer physical burdens	7 (10.4)	11 (16.4)	7 (10.4)	19 (28.4)	23 (34.3)
Indispensable Immunosuppressant	25 (37.3)	8 (11.9)	2 (3.0)	1 (1.5)	30 (44.8)
Performed positively abroad	18 (26.9)	16 (23.9)	6 (9.0)	5 (7.5)	22 (32.8)
Only for poor control patients	18 (26.9)	11 (16.4)	7 (10.4)	25 (37.3)	6 (9.0)
Complication is severe	11 (16.4)	7 (10.4)	10 (14.9)	3 (4.5)	36 (53.7)
Possible at any large hospital	0 ( 0.0)	6 (9.0)	7 (10.4)	39 (58.2)	14 (20.9)
Become insulin-free	26 (38.8)	22 (32.8)	9 (13.4)	3 (4.5)	7 (10.4)

Item description was abbreviated.

^(1)^A: correct, B: wrong, C: not always correct.

**Table 4:** Factors related to knowledge about pancreatic islet transplantation (*N* = 137; logistic simple linear regression analysis).

Variable	Knew islet transplantation		
	OR	[95% CI]	
Gender (female versus male)	1.15	[0.59–2.25]	
Age	0.99	[0.96–1.01]	
Education (≥12 years versus <12 years)	2.45	[1.23–4.88]	*
Body mass index	0.92	[0.82–1.03]	
Onset of diabetes (age)	0.99	[0.99–1.04]	
Duration of diabetes (years)	1.00	[0.97–1.04]	
Diabetic complication			
Neuropathy	1.24	[0.58–2.64]	
Retinopathy	0.73	[0.34–1.56]	
Nephropathy	0.53	[0.18–1.52]	
HbA1c	0.87	[0.66–1.16]	
SMBG	1.26	[1.04–1.52]	*
Hypoglycemic episode (yes versus none)	4.66	[1.75–12.4]	**
Hypoglycemic episode (times)	0.78	[0.60–1.01]	
Current treatment (CSII versus injection)	15.1	[1.90–119]	*

OR: odds ratio, CI: confidence interval *P < 0.05  **P < 0.01 when OR is more than 1, it is assumed that patients knew about islet transplantation.
